# Regional and Local Inequalities in Disability Status by Sexual Orientation and Gender Identity: A Cross-Sectional Ecological Analysis of the 2021 Census of England and Wales

**DOI:** 10.1089/heq.2023.0231

**Published:** 2024-04-15

**Authors:** Robert J. Romanelli

**Affiliations:** RAND Europe, Health and Wellbeing Research Group, Cambridge, United Kingdom.

**Keywords:** LGBT, inequalities, disparities, disability, socioeconomic deprivation, urbanization

## Abstract

**Purpose:**

To examine regional differences in disability status by sexual orientation and gender identity and to explore local factors that are associated with levels of inequalities for people who identify as lesbian, gay, bisexual, or other sexual orientations (LGB+) or transgender.

**Methods:**

This was a cross-sectional ecological analysis of 2021 Census data from England and Wales. The main outcome variable was disability status. The main explanatory variables were sexual orientation and gender identity. Weighed linear regression was used to examine differences in disability status by sexual orientation (LGB+ vs. heterosexual) and gender identity (transgender vs. cisgender). The magnitude of between-group differences was explored by region and, in England, local authority-level urbanization and socioeconomic deprivation.

**Results:**

Among 48.5 million census respondents within 331 local authority districts (LADs) across England and Wales, LGB+ and transgender groups were more likely to report having a disability than their heterosexual and cisgender counterparts. Inequalities were prevalent across regions of England and Wales, but were smallest in the Greater London area and largest in the southwest of England. Inequalities were also larger within English LADs that were relatively less urbanized and relatively more socioeconomically deprived.

**Conclusions:**

This study identified disparities in disability status by sexual orientation and gender identity, which varied by region and local socioeconomic deprivation and urbanization. More research is needed to better understand how to support disabled LGBT+ people, especially those in less urbanized and more socioeconomically deprived areas.

## Introduction

Approximately 16 million people in the United Kingdom (UK), or ∼24% of the population in 2021/22 had a disability,^1^ defined as an illness or condition lasting at least 12 months that limits the ability to carry out day-to-day activities. Disabled people face numerous inequalities in the UK. For example, they are less likely to obtain educational qualifications and more likely to be unemployed.^2^ Furthermore, households with a disabled adult have significantly lower median incomes than households without a disabled adult, and they are more likely to live in poverty.^3^ Lastly, disabled people more frequently report lower levels of happiness and life satisfaction and higher levels of loneliness and anxiety,^2^ and they are more likely to be a victim of a crime and domestic violence.^4^

A recent analysis of the 2021 Census data from England and Wales by RAND Europe has revealed a higher prevalence of disability among people who identify as lesbian, gay, bisexual, transgender, or other sexual orientations or gender identities (LGBT+) than those who are heterosexual or cisgender.^5^ Specifically, lesbian, gay, bisexual people and those of other sexual orientations, such as pansexual or asexual, (LGB+) and people whose gender identity is different from the sex assigned to them at birth (i.e., transgender) are, respectively, 47% and 41% more likely to report having a disability compared with their heterosexual and cisgender counterparts.

This is a striking difference considering that the age distribution of LGBT+ census respondents skews much younger than non-LGBT+ respondents.^5^ The analysis further found that LGBT+ people are also more likely to face other inequalities, such as poorer self-reported general health, lower employment levels, and, among transgender people specifically, lower levels of educational qualifications; however, the analysis could not determine whether worse outcomes were related to disability status.^5^

LGBT+ people and disabled people each encounter social stigma and systemic discrimination in their day-to-day lives, which can negatively impact well-being, physical and mental health, and exacerbate minority stress.^6–9^ The intersectionality of being LGBT+ and disabled can further impact experiences of stigma and discrimination and its downstream consequences.^10,11^

In 2021, The census of England and Wales introduced voluntary questions on sexual orientation and gender identity for equity monitoring under the UK's Equality Act 2010 and to inform appropriate resource allocation.^12^ A deeper understanding of where in England and Wales LGBT+ people disproportionately have a disability compared with non-LGBT+ people is needed to develop policies to address inequalities in this doubly marginalized group.

This study was conducted to examine regional variation in LGBT+ inequalities related to disability status in England and Wales and to explore the association between local geographic factors, specifically urbanization and socioeconomic deprivation, and the magnitude of inequalities. Findings from this study can help to advance commitments made by local governments in the UK to improve the lives of LGBT+ people and to help inform future iterations of the UK's National Disability Strategy.^13–16^

## Methods

### Study design

This was an ecological cross-sectional analysis of anonymized data from the 2021 Census of England and Wales. Owing to the anonymized nature of the data, research ethics approval was not required.

Publicly available data files were downloaded from the UK's Office of National Statistics (ONS).^17^ Data files included sexual orientation and gender identity by age, disability, ethnicity, and sex, with observations aggregated at the sexual orientation or gender identity response level by responses to the other variables for each local authority district (LAD). The census includes 331 LADs (309 in England and 22 in Wales).

### Outcome

The primary outcome was disability status. The census asked participants: “Do you have limitations to daily activities due to an illness or condition lasting, or expecting to last, 12 months or more?” Responses were coded as: “no”; “a little”; or “a lot.” Individuals who responded “a little” or “a lot” were categorized as disabled, consistent with the definition of disability in the UK's Equality Act 2010.^18^

### Explanatory variables

The main explanatory variables were sexual orientation and gender identity. Census participants were asked, “Which of the following best describes your sexual orientation?” and “Is the gender you identify with the same as your sex registered at birth?” as voluntary questions among individuals 16 years of age or older. Responses for sexual orientation were coded as “straight or heterosexual,” “gay or lesbian,” or “other sexual orientation,” with an option to write in self-described sexual orientation (e.g., bisexual or pansexual). Responses for gender identity were coded as “yes” or “no,” with an option to write in self-described gender identity (e.g., nonbinary or gender nonconforming).

For this analysis, respondents were categorized as “heterosexual” if they reported their sexual orientation as “heterosexual or straight” or as “LGB+” if they reported their sexual orientation as “gay or lesbian” or “other sexual orientation” (bisexual was the most commonly self-reported “other sexual orientation,” hence the inclusion of “B”).^19^ Respondents were categorized as “cisgender” if they reported their gender identity was the same as the sex assigned at birth or “transgender” if they reported their gender identity was not the same as the sex assigned at birth.

As these questions were voluntary, individuals who did not offer a response were categorized as “nonresponders.” For each sexual orientation and gender identity group by LAD, the percentage of respondents who were <35 years of age, 65 years of age or older, female, or of nonwhite ethnicity was calculated. LADs were further grouped into 10 regions: East Midlands, West Midlands, east of England, Greater London Area, northeast, northwest, southeast, southwest, Yorkshire and The Humber, and Wales.

Additional LAD characteristics for England (2011 rural–urban classification and 2019 indices of multiple deprivation [IMD]) were downloaded from the ONS website.^20,21^ IMD are a composite weighted socioeconomic metric based on several domains of deprivation: income (22.5%); employment (22.5%); education, skills, and training (13.5%); crime (9.3%); barriers to housing and services (9.3%); and living environment (9.3%).^20^ Small statistical areas in England (*N*=32,844) are ranked from 1st (most deprived) to 32,844th (least deprived). This ranking can be aggregated at the level of the LAD by averaging the ranks of small statistical areas within each LAD. For this study, average IMD ranks were expressed as deciles, with 1st representing the most deprived and 10th representing the least deprived.

Rural–urban classification was categorized as “mostly rural,” “largely rural,” “urban with significant rural,” “urban with city and town,” or “urban with conurbation,” based on standard definitions.^21^ Wales was excluded from this analysis, as these data were not available at the LAD level.

### Statistical analysis

Weighted ordinary least square (OLS) linear regression was used to compare differences in the percentage of census participants reporting a disability across LADs by sexual orientation and gender identity. The unit of observation was sexual orientation response (*N*=3; LGB+, heterosexual, or nonresponder) or gender identity response (*N*=3; transgender, cisgender, or nonresponder) by LAD (*N*=331), with a total of 993 observations, each. Regression weights represented the total number of respondents at the unit of observation. Hereafter units are referred to as “groups.” Standard errors were calculated using robust variance–covariance estimation by LAD.

Sexual orientation and gender identity variables were each regressed on the outcome of percentage of respondents who reported having a disability, before and after statistical adjustment for group-level age, sex, ethnicity, and region to arrive at between-group percentage-point differences in disability status with 95% confidence intervals (CIs). From these models, the marginal mean percentages of respondents with a disability by sexual orientation and gender identity after statistical adjustment were also generated.

Weighted OLS models were also used to examine differences in disability status for sexual orientation or gender identity groups stratified by region, urbanization, and socioeconomic deprivation. Percentage-point differences were generated with 95% CI, before and after statistical adjustment for group-level sex, age, and ethnicity.

For all analyses, statistical significance was set at an alpha of 0.05. All analyses were conducted in Stata 17.0.

## Results

### Population characteristics

Among ∼48.6 million respondents from the 2021 Census of England and Wales, 3.2% are LGB+ and 0.5% are transgender. Compared with heterosexual census respondents, LGB+ respondents are disproportionately younger (57.9% vs. 28.4% 16 to 34 years of age) but are largely similar on other characteristics (Table 1). Nearly one in five (19.8%) LGB+ respondents reside in the Greater London area compared with 14.1% of heterosexual respondents. Transgender respondents are also disproportionately younger than cisgender respondents (47.6% vs. 29.4% 16–34 years of age) and are disproportionately nonwhite (38% vs. 16%).

**Table 1.** England and Wales Census Participant Characteristics by Sexual Orientation and Gender Identity

	Sexual orientation (%)			Gender identity (%)		
	LGB+ (***N***=1,536,623)	Heterosexual (***N***=43,403,128)	Not answered (***N***=3,626,636)	Transgender (***N***=262,121)	Cisgender (***N***=45,389,644)	Not answered (***N***=2,914,609)
Age, years						
16–34	57.9	28.4	32.3	47.6	29.4	31.4
35–64	37.7	48.5	40.9	42.5	48.0	42.1
65 and older	4.4	23.1	26.8	9.8	22.6	26.5
Sex						
Male	45.9	48.6	47.7	50.1	48.3	50.3
Female	54.1	51.4	52.3	49.9	51.7	49.7
Ethnicity						
Asian	5.2	8.5	12.0	15.7	8.3	13.4
Black	2.3	3.7	4.8	11.1	3.6	5.2
Multiple	4.0	1.9	2.6	3.5	2.0	2.4
Other	2.1	1.9	3.0	7.3	1.9	3.3
White	86.4	84.0	77.7	62.5	84.3	75.7
Region						
East Midlands	7.4	8.3	8.1	7.4	8.2	8.3
East of England	8.9	10.7	10.2	9.1	10.6	9.9
Greater London	19.8	14.1	18.6	24.6	14.3	19.2
Northeast	4.3	4.6	3.6	3.8	4.6	3.5
Northwest	12.5	12.5	11.1	11.4	12.5	11.0
Southeast	15.3	15.6	14.7	13.3	15.7	14.1
Southwest	9.8	9.8	9.5	7.6	9.8	9.1
West Midlands	8.0	10.0	10.0	9.8	9.8	10.4
Yorkshire and The Humber	8.9	9.2	8.8	9.1	9.2	8.9
Wales	5.0	5.3	5.4	3.9	5.3	5.6

LGB+, lesbian, gay, bisexual, or other sexual orientation.

Nearly one-quarter (24.6%) of transgender respondents reside in the Greater London area compared with 14.3% of cisgender respondents. Nonresponders to questions on sexual orientation and gender identity tend to fall between heterosexual and cisgender groups, respectively, on the distribution of most characteristics. In England, LGB+ compared with heterosexual respondents and transgender compared with cisgender respondents more frequently reside in urban LADs with conurbation and in LADs that fall, on average, among the 10% least socioeconomically deprived (Table 2).

**Table 2.** English Local Authority Characteristics by Sexual Orientation and Gender Identity

	Sexual orientation (%)			Gender identity (%)		
	LGB+ (***N***=1,379,299)	Heterosexual (***N***=38,351,912)	Not answered (***N***=3,220,172)	Transgender (***N***=239,666)	Cisgender (***N***=40,125,328)	Not answered (***N***=2,586,339)
Urban–rural classification						
Mostly rural	6.5	9.0	8.0	4.7	8.9	7.5
Largely rural	8.6	11.6	10.2	6.8	11.5	9.8
Urban with significant rural	10.0	12.5	10.8	8.3	12.5	10.4
Urban with city and town	28.4	26.1	25.9	26.9	26.1	26.0
Urban with conurbation	46.5	40.8	45.1	53.3	40.9	46.4

	***N* =1,433,545**	***N* =40,141,848**	***N* =3,355,498**	***N* =246,807**	***N* =41,993,364**	***N* =2,690,683**
Average IMD rank decile						
1 (most deprived)	5.3	7.1	6.0	4.1	7.0	5.6
2	6.2	7.6	6.7	4.7	7.5	6.3
3	6.6	8.7	7.6	5.6	8.6	7.2
4	7.5	8.4	8.3	7.1	8.4	8.1
5	9.2	10.1	10.0	8.7	10.1	9.9
6	9.4	8.7	8.7	8.1	8.8	9.5
7	9.0	9.8	9.6	9.5	9.8	9.6
8	14.2	13.4	13.4	13.9	13.4	13.4
9	14.6	11.9	13.0	16.8	11.9	13.4
10 (least deprived)	17.8	14.2	16.8	21.4	14.3	17.8

IMD, indices of multiple deprivation.

### Differences in disability status by sexual orientation and gender identity

In unadjusted analysis, LGB+ groups more frequently report being disabled than the heterosexual groups (Table 3). Adjusting for group-level age, sex, ethnicity, and region increases differences in disability status between LGB+ and heterosexual groups relative to the unadjusted differences. Smaller, but statistically significant, differences in disability status are observed between nonresponders to the sexual orientation question and heterosexual groups. Similar results are observed by gender identity; although the difference in disability status also increases after statistical adjustment for transgender groups versus cisgender groups, the magnitude of the adjusted disparity is smaller (+12.6 percentage points vs. cisgender group) than that of the LGB+ group (+17.7 percentage points vs. heterosexual group).

**Table 3.** Disability Status by Sexual Orientation and Gender Identity

	Disability, % raw value	Unadjusted difference (95% CI)	Disability, % marginal value	Adjusted difference^*^ (95% CI)
Sexual orientation				
Heterosexual	19.5	Ref	19.2	Ref
LGB+	28.8	9.3 (8.7–9.9)^***^	36.9	17.7 (15.3–20.0)^***^
Not answered	23.3	3.8 (3.4–4.1)^***^	23.2	4.0 (3.6–4.4)^***^
Gender identity				
Cisgender	19.9	Ref	19.9	Ref
Transgender	28.1	8.2 (6.9–9.4)^***^	32.5	12.6 (11.4–13.8)^***^
Not answered	22.2	2.3 (1.9–2.7)^***^	21.7	1.8 (1.1–2.5)^***^

^*^ Models adjusted for region and observation-level age, sex, and ethnicity; ^***^p<0.001.

### Regional differences in disability status by sexual orientation

**FIG. 1.**
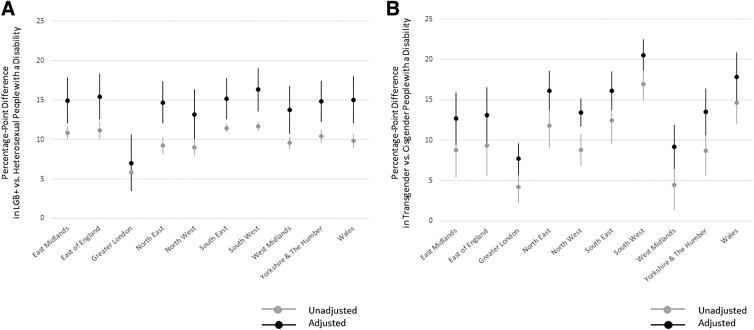
Disability status by sexual orientation/gender identity and region. Point estimates derived from weighted OLS linear regression models with statistical adjustment for sexual orientation or gender identity response-level age, sex, and ethnicity, rural–urban classification, and socioeconomic deprivation. *Error bars* represent 95% CIs. **(A)** Percentage-point differences by sexual orientation. **(B)** Percentage-point differences by gender identity. CI, confidence interval; LGB+, lesbian, gay, bisexual, or other sexual orientation.

LGBT+ groups are, on average, more frequently disabled across regions, both before and after statistical adjustment for other factors (Fig. 1). The Greater London area has the lowest levels of inequality related to disability by sexual orientation, compared with other regions, but LGB+ groups in London are still more likely to report being disabled than heterosexual groups (adjusted difference=+7.0 percentage points; 95% CI: 3.5–10.6; Fig. 1A). Between-region variation in LGB+ inequalities is relatively small across the other regions, ranging from +13.2 percentage points in the northwest to +16.3 in the southwest, after statistical adjustment.

### Regional differences in disability status by gender identity

Similarly, the Greater London area has the lowest levels of inequality related to disability by gender identity compared with other regions, but transgender groups in London are still more likely to report having a disability than cisgender groups (adjusted difference=+7.7 percentage points; 95% CI: 5.8–9.6; Fig. 1B). Between-region variation in transgender inequalities related to disability is relatively wide across other regions, ranging from +9.2 percentage points in the West Midlands to +20.5 in the southwest, after statistical adjustment.

### Urbanization and disability status

**FIG. 2.**
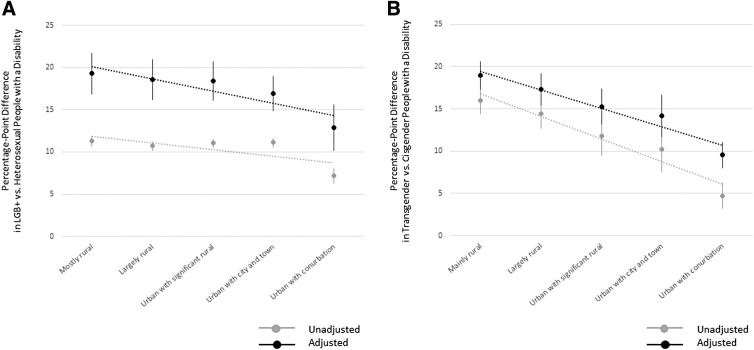
Disability status by sexual orientation/gender identity and local authority district urban–rural classification in England. Point estimates derived from weighted OLS linear regression models with statistical adjustment for sexual orientation or gender identity response-level age, sex, and ethnicity, region, and socioeconomic deprivation. *Error bars* represent 95% CIs. *Dotted lines* represent linear trends. **(A)** Percentage-point differences by sexual orientation. **(B)** Percentage-point differences by gender identity.

LGBT+ inequalities related to disability status in England are larger among LADs that are less urbanized compared with those that are more urbanized, before and after statistical adjustment for other factors (Fig. 2). Linear trends by urbanization are weaker for LGB+ groups (Fig. 2A) compared with transgender groups (Fig. 2B), based on regression slopes.

### Socioeconomic deprivation and disability status

**FIG. 3.**
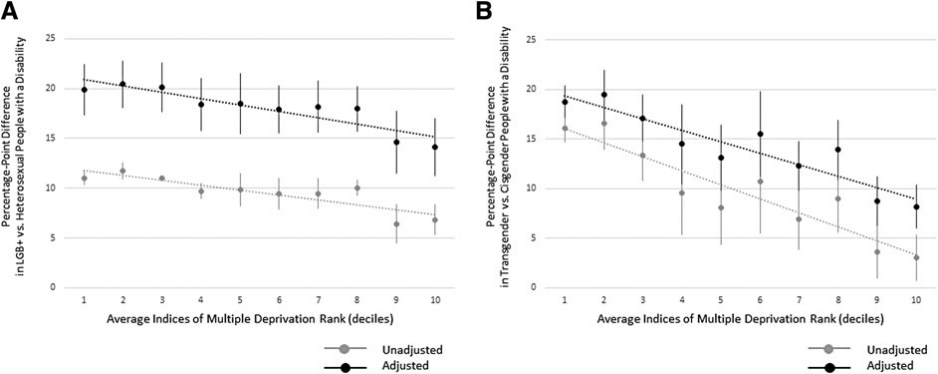
Disability status by sexual orientation/gender identity and local authority district socioeconomic deprivation in England. Point estimates derived from weighted OLS linear regression models with statistical adjustment for sexual orientation or gender identity response level age, sex, and ethnicity, region, and rural–urban classification. *Error bars* represent 95% CIs. *Dotted lines* represent linear trends. **(A)** Percentage-point differences by sexual orientation. **(B)** Percentage-point differences by gender identity.

LGBT+ inequalities related to disability status in England are larger among LADs with higher levels of socioeconomic deprivation (i.e., lower average IMD rank; Fig. 3). Linear trends by socioeconomic deprivation are weaker for LGB+ groups (Fig. 3A) than for transgender groups (Fig. 3B), based on regression slopes.

## Discussion

This cross-sectional analysis of data from the 2021 Census of England and Wales points to LGBT+ inequalities related to disability status, such that higher percentages of LGB+ and transgender respondents report being disabled than their heterosexual and cisgender counterparts, both before and after adjusting for group-level differences in age, sex, ethnicity, and region. Inequalities related to disability status are prevalent across regions, and regional variation in inequalities is wider for transgender than for LGB+ groups, but for both the Greater London area has the smallest levels of inequalities, whereas the southwest has the largest.

In England, LGBT+ inequalities are generally wider in LADs that are less urbanized (i.e., more rural) and have higher levels of socioeconomic deprivation. To our knowledge, this is the first study to examine inequalities in disability status among LGBT+ versus non-LGBT+ groups in the UK and to explore regional and local variation in the prevalence of such inequalities. Findings could help to identify areas in England and Wales that may have greater needs for policy and resources to address disparities.

Overall, although about one in five heterosexual and cisgender people from the census of England and Wales report having a disability, these figures are 34.5% and 30.0% for LGB+ and transgender people, respectively, after adjusting for other factors. In an analysis of the general practice (GP) survey from England, 2021, Saunders et al. reported higher odds of a learning disability, deafness or hearing loss (which could be classified as a disability under the UK's Equality Act), or “other long-term condition or disability” among trans and nonbinary respondents compared with cisgender respondents registered with a GP.^22^

Saunders et al. also previously reported that LGB+ individuals compared with heterosexual individuals had higher odds of deafness or hearing loss among women of any age or among people 18–35 years of age across gender, using data from the GP survey from England, 2015 to 2017.^23^ We are unaware of other studies from the UK that have examined disability status more broadly among LGBT+ versus non-LGBT+ groups.

In the United States, a study conducted by Fredricksen-Goldsen et al. found higher rates of disability among LGB individuals than their heterosexual peers from the Washington State Behavioral Risk Factor Surveillance System, 2003–2009.^24^ Another study from a California cohort between 2004 and 2005 also showed higher rates of disability, defined as “reports of functional health limitations” in LGB individuals, than heterosexual individuals, but much of these effects were nullified when adjusting for other medical conditions.^25^

The cause of disabilities in this study is unknown. A research briefing on disabilities in the UK published in August 2023 for the House of Commons indicates that the most common impairments reported by people with disabilities include mobility (47%), stamina/breathing/fatigue (35%), and mental health (32%).^1^ Over the past decade, rates of mental health-related disabilities have been proportionally rising, whereas mobility-related disabilities have been declining, and other types of disabilities have remained relatively constant.^1^

One hypothesis is that the higher prevalence of disability in LGBT+ people is due to mental health conditions. Numerous studies describe a higher burden of depression, anxiety, self-harm, and suicide ideation and attempts in this population than the cisgender heterosexual population, as a result of experiences of social stigma, internalized homophobia, biphobia, and transphobia, and discrimination.^26–30^

In the context of the above-mentioned mental health hypothesis as the potential driver of these inequalities, LGBT+ people in Greater London, a large and dense urban center, may have better community and psychosocial support than less urbanized regions, leading to better mental health outcomes. This is consistent with the findings that, at the local level, larger disparities are observed, on average, within LADs that are less urbanized.

Independent of urbanization, more socioeconomically deprived LADs also have on average large disparities in disability for LGBT+ people than less socioeconomically deprived LADs. A qualitative study from Scottland found that the experiences of LGBT+ people living in socioeconomically deprived areas “were often tinged with a fear that they might be victims of hate crime or aggression.”^31^ Furthermore, negative experiences extended to external interactions, with LGBT+ participants from the study describing having to navigate the stigma associated with living in more socioeconomically deprived areas and the misconception that they are “sponging off society.”

Accordingly, LGBT+ people living in more socioeconomically deprived or less urbanized areas may face more social stigma and discrimination and may have higher levels of stress, less psychosocial support, and fewer connections with other LGBT+ people. They also may be more likely to be “closeted” completely or in certain social settings, such as work or school. Together, these factors could lead to worse mental health outcomes than LGBT+ people living in less deprived and/or more urbanized areas. For LGBT+ people living in socioeconomically deprived areas, the stigma of being LGBT+ may be compounded by the stigma and economic stress associated with poverty, further exacerbating physical and mental health issues and, consequently, leading to disability.^32^

Lastly, disabled people also experience minority stress, related to stigma and discrimination, which could impact on their physical and mental health.^6^ Thus, disabled LGBT+ people likely experience multiple levels of stigma and discrimination related to their sexual orientation, gender identity, and disability status, which may be further amplified by poverty-based stigma.

The impact of long-term physical conditions on LGBT+ disability should also be considered, as prior work from England shows that LGBT+ groups are more likely to have long-term physical conditions than cisgender heterosexual groups.^22,23^ Such conditions could lead to disability. Regardless of the cause, living with a disability might impact migration patterns, potentially explaining the disproportionate burden of disability among LGBT+ people living in less urbanized and more socioeconomically deprived areas.

Many LGBT+ people born in less urbanized or more deprived areas tend to migrate to urban centers, such as London and Manchester, as they enter adulthood, where they can build a support network or “chosen family.”^33^ Nevertheless, disabled LGBT+ people may be less likely to migrate due reliance on family or other informal caregivers in the villages in which they were born and raised.

Future research is required to test these hypotheses to identify the drivers of a higher prevalence of disability in LGBT+ people, as well as to better understand the life experiences of disabled LGBT+ people and their needs. Meanwhile, policy makers in the UK should consider how the National Disability Strategy can better support disabled LGBT+ people, as there is little mention of this population in the current strategy document.

This study has several limitations that should be considered when interpreting the findings. This is an observational study and causal inferences cannot be made. The study was conducted on data aggregated at the level of sexual orientation and gender identity groups by LAD, and observed associations between region, socioeconomic deprivation, and rural–urban classification are not at the level of the individual and should be interpreted with caution.

Although statistical adjustment was conducted for several factors that may explain the relationship between sexual orientation or gender identity and regional and local variation in disability status, such methods on aggregated data might not have the same impact as they do on individual-level data, potentially resulting in biased estimates. The aggregated nature of the data also preluded the ability to conduct stratified analysis by other characteristics (e.g., sex or ethnicity).

Other limitations of this study relate to how the census data were collected, such as the manner in which sexual orientation and gender identity status were operationalized (e.g., exclusion of commonly used terms, such as “queer,” “bisexual,” or “gender nonconforming”). Given issues with these categories and historical mistrust between LGBT+ communities and the UK government, it is likely that the census has underestimated LGBT+ people.^5^

Similarly, disabled people may have been reluctant to disclose their disability due to stigma or internalized ableism. Furthermore, the census does not provide more granular information on the nature of disabilities (e.g., physical, mental, or learning). Thus, we cannot truly know what is driving differences in the observed disparities between LGBT+ and non-LGBT+ populations.

Despite these limitations, this study has several strengths. It was conducted on a large representative population of England and Wales, leveraging multiple publicly available data sources. Furthermore, it has generated several testable hypotheses for the observed higher prevalence of disability among sexual orientation or gender identity minority groups that can inform policy decision making.

## Conclusions

In summary, the 2021 Census of England and Wales reveals disparities in disability status by sexual orientation and gender identity, which vary by region and local socioeconomic deprivation and urbanization. More research is needed to better understand how to better support disabled LGBT+ people, especially those in less urbanized and more socioeconomical deprived areas.

## Abbreviations Used

CI: confidence interval
GP: general practice
IMD: indices of multiple deprivation
LAD: local authority district
LGB+: lesbian, gay, bisexual, or other sexual orientations
LGBT+: lesbian, gay, bisexual, transgender, or other sexual orientations or gender identities
OLS: ordinary least square
ONS: Office of National Statistics
